# Large-Scale Evaluation of Molecular Descriptors by Means of Clustering

**DOI:** 10.1371/journal.pone.0083956

**Published:** 2013-12-31

**Authors:** Matthias Dehmer, Frank Emmert-Streib, Shailesh Tripathi

**Affiliations:** 1 UMIT, Division for Bioinformatics and Translational Research, Eduard Wallnoefer Zentrum 1, Hall in Tyrol, Austria; 2 Queen's University Belfast, Computational Biology and Machine Learning, Center for Cancer Research and Cell Biology, School of Medicine, Dentistry and Biomedical Sciences, Belfast, United Kingdom; Jacobs University Bremen, Germany

## Abstract

Molecular descriptors have been explored extensively. From these studies, it is known that a large number of descriptors are strongly correlated and capture similar characteristics of molecules. In this paper, we evaluate 919 Dragon-descriptors of 6 different categories by means of clustering. Also, we analyze these different categories of descriptors also find a subset of descriptors which are least correlated among each other and, hence, characterize molecular graphs distinctively.

## Introduction

Molecular descriptors map molecular structures to the reals by taking physical, chemical or structural information into account [1]. A large number of descriptors have been developed to describe different properties of molecular graphs. Therefore, these descriptors can be classified into different categories depending what kind of information is used (e.g., physical, chemical or structural information) to define such a measure. The commercial software package Dragon [2] (version 6.0.26) contains 4885 molecular descriptors which are classified into 29 categories.

The problem of analyzing molecular descriptors by applying clustering techniques has been already explored [3]–[6]. These are usually based on using principal component analysis (PCA) and correlation-based methods for the identification of different descriptors. For example, Todeschini et al. [6] and Basak et al. [3] evaluated descriptors on a rather small collection of molecular graphs using PCA and ranked them based on the intercorrelation. In order to find similarities between molecular descriptors, Basak et al. [4], [5] used a PCA-based clustering technique on both a hydrocarbon dataset and mixed chemical compounds. Taraviras et al. [7] performed a cluster analysis with 240 descriptors by using different clustering algorithms. The weak point of the just sketched approaches is that the corresponding study has not been performed on a large scale (large data sets) and with distinct descriptors belonging to several categories. Also, the optimal number of different descriptors (dimension) has not been validated statistically. In this paper, we overcome these problems.

A thorough evaluation of the vast amount of developed descriptors [1] is required to identify categories of descriptors which capture structural information differently. In our analysis we evaluate 6 categories (see next section) of structural descriptors by means of clustering. The main contribution of this paper is to explore the *dimension* of the descriptor space, i.e., how many different descriptors exist among all which have been introduced so far. Here, we put the emphasis on 919 structural descriptors from Dragon. In particular, we find that only a very few descriptors are different. In this context that means they are least correlated and, therefore, capture structural information differently.

## Methods and Results

### Molecular Descriptors

To perform our study, we used six categories of descriptors implemented in Dragon (version 6.0.26) which are defined as follows:

1. **Connectivity indices** [1]: These indices are calculated from the vertex-degree of a molecular graph. The Randić index [8] is a prominent example thereof.
2. **Edge adjacency indices** [1]: These indices are based on the edge adjacency matrix of a graph. The resulting descriptor-value is the sum of all edge entries of the adjacency matrix of a graph. Balaban et al. [9] developed several indices by using graph-theoretical matrices.
3. **Topological indices** [1]: These structural graph measures which take various structural features into account, e.g., distances and eigenvalues. The term *topological index* has been firstly coined by Hosoya [10]. The first and the second Zagreb indices [11] are prominent examples thereof.
4. **Walk path counts** [1]: These indices are defined by counting paths or walks of a graph. Here, the term *walk* refers to random walks which is based on using a probability measure. We point out that such indices have been listed by Todeschini and Consonni [1].
5. **Information indices** [1]: These measures are based on using Shannon's entropy. To assign a probability value to a graph, Dragon uses so-called partition-based methods [12] by using several graph invariants such as vertices, edges, vertex degrees and distances have been used [12]. The so-called topological information content [13] and the Bonchev-Trinajstić index [14] are prominent examples of partition-based information indices. So-called partition-independent information-theoretic measures for graphs have been developed by Dehmer [12].
6. **2D Matrix-based** [1]: These descriptors are calculated based on the elements of so-called graph-theoretical matrices [15] by using several algebraic operations. The Balaban-like indices inferred from the adjacency matrix [2], [9] are important examples of this category.

We want to emphasize that the term 'Topological indices' is here misleading and ambiguous. For example, typical information indices are based on structural features of a graph by using Shannon's entropy. So, they represent topological indices too. The same holds for all other groups which have been defined by using structural features of molecular structures and, therefore, they are topological indices as well, see [1], [9], [16]–[19].

### Data

In order to evaluate the above mentioned 6 categories of descriptors, we use 3 data sets namely:

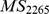
 contains (non-isomorphic) molecular structures (only skeletons, i.e., without vertex- and edge labels) inferred from the NIST spectral database [20].

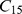
 contains exhaustively generated (non-isomorphic) tree structures with 15 vertices each [20].

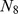
 contains exhaustively generated (non-isomorphic) graphs with 8 vertices each [21].

To perform our analysis, we calculate the descriptor values for these three datasets. We removed those descriptors which give constant and erroneous values by using the three data sets. The erroneous values are produced by those descriptors for which we have not been able to calculate a descriptor value of a network without additional physical or chemical information. Finally, we the above mentioned six categories contain 24, 301, 57, 28, 40, 469 descriptors.

### Clustering Techniques

Clustering is an unsupervised learning technique which aims to find different groups or clusters of objects in data [22]. The groups are described as a collection of objects which are closer to each other than the rest of the objects [22]. An example thereof is hierarchical clustering as groups of the objects are arranged in a hierarchical order by a so-called dendogram. The objects which are clustered in one group have a higher degree of similarity than the objects which are clustered in different groups. Thus a resulting clustering solution allows to determine clusters where each cluster shows distinct property of the data. The similarity or dissimilarity between two objects is usually determined by using a Similarity/distance function which measures the similarity/distance between data points of different objects. Examples are the Euclidean distance, the Manhattan distance or the correlation-based distance. A dissimilarity can be described as follows:

Several algorithms have been developed for cluster analysis [22]. These algorithms can be divided into several categories namely partition-based clustering, hierarchical clustering, density-based clustering, grid-based clustering and fuzzy clustering [22], [23]. Thus k-means, soft k-means Clustering, k-medoids Clustering [22] are some examples representing non-hierarchical clustering methods. Hierarchical clustering itself can be divided into two categories called agglomerative and divisive clustering [22]. As known, several concrete methods thereof have been developed such as single linkage, complete linkage and average linkage, see [22].

In order to evaluate the descriptors, we perform hierarchical clustering (average linkage) by using the mentioned Dragon descriptors and the Spearman rank correlation as a distance measure. Here, we denote the correlation matrix between descriptors as 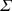. Then, the distance between a pair of descriptors is defined by.
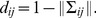
(1)


In order to choose a clustering method we use the cophenetic correlation measure [24]. A high correlation coefficient shows that the distance between the data points is well preserved by the created dendogram of the hierarchical clustering solution. In our analysis, the cophenetic correlation coefficient is highest for the average clustering solution for all three data-set compared to other clustering algorithms. We calculate the cophenetic correlation for seven hierarchical clustering algorithms which are the Ward, Single, Complete, Average, Mcquitty, Median and the Centroid-method. The cophentic correlation coefficients for the average clustering solutions for three data-sets are 0.84, 0.89 and 0.93.

### Cluster Validity

Cluster validity [23], [25] is used to evaluate the quality of clustering solution (by using a certain clustering algorithm), e.g., the optimum number of clusters in the data, or whether the resulting cluster solution fits the data. Known clustering validation techniques are divided into three categories namely internal, external and relative validity criteria. External validation criteria evaluate clustering solutions with a predefined clustering structure. Using internal validation criteria relates to find the optimal number of clusters which is based on the intrinsic knowledge of data. Relative validation criteria are used to compare two different clustering solutions [23].

In order to perform analyses, we use external and internal clustering validation criteria. For the external validation, we compared the clustering solution with a predefined group of clusters which serve as reference clusters. The external clustering validity of a clustering solution with respect to the given reference cluster is estimated by using the information-theoretic quantity 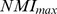 (normalized mutual information) [26], [27] defined by
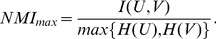
(2)where



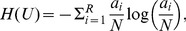
(3)

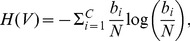
(4)

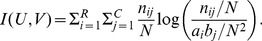
(5)


Hereby, we assume that we have two clustering solutions 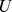 and 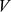 which have 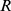 and 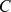 clusters. The overlap between these two clusters is shown in the contingency Table 1. We calculated 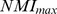 for all three data-sets with different number of clusters.

**Table 1:** A contingency table which defines the overlap between two cluster solutions, 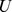 and 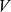.

U 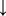 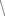 V 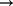	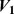	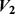	.	.	.	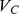	Sums
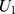	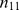	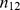	.	.	.	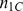	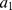
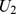	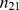	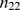	.	.	.	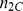	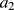
.	.	.	.	.	.	.	.
.	.	.	.	.	.	.	.
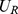	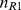	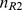	.	.	.	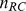	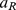
Sums	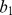	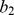	.	.	.	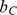	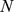

### The Optimal Number of Clusters

The optimal number of clusters (internal cluster validity) are determined by consensus clustering [27], [28] which has been here performed as follows. Assume we evaluate 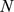 descriptors on a dataset containing 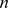 molecular graphs. Thus we get 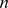 descriptor values for each descriptor. First, we resample the data of sample-size, 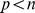, 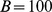 times for 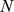 descriptors to generate 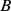 clustering solutions 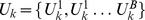, for 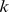 clusters, where 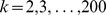. After that we calculate the consensus indices for each cluster, 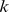, which is defined as follows:
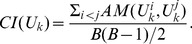
(6)


As to the measure 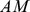, we use the adjusted rand index 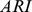 [29] defined by.
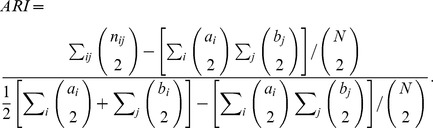
(7)


The number of clusters 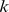 for which 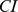 attains its maximum is chosen as the optimal number of clusters, namely.
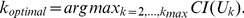
(8)


### Determining a Highly Correlated Subset of Descriptors

Let 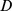 be a set of descriptors and 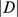 is its cardinality. Let 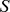 be a subset of 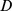. The selected 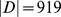 descriptors can be reduced to a set of descriptors, 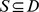. The remaining 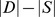 descriptors will have a significant correlation with at least one of the descriptor in the set 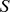 and the descriptors in 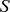 are not significantly correlated. If two descriptors are showing a significant correlation with each other, then we conclude that they capture structural information similarly. In order to predict the significance of the correlation between two descriptors, we perform the following approach:

Let 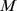 be a dataset of 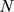 descriptors and 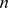 samples. First, we generate bootstrap datasets, 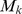, 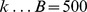 possessing sample size 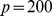, where 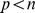. Then, for each dataset, 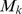, we perform a correlation test [30], [31] between each pair of descriptors and obtained a *p value* 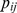 for each pair. Thus, we test 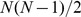 hypotheses for all pairs. In order to control the false positives in the multiple hypothesis testing problem, we use the *bonferroni correction* method for multiple testing correction (MTC) [32] and obtained adjusted *p-values*. For each pair these adjusted *p-values* are denoted by 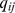. In order to decide whether the correlation between a pair is significant, we choose 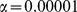. After applying the correlation test and MTC, we obtain a binary matrix 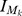 which is defined follows:
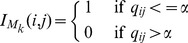
(9)


Finally we calculate a summary-statistic, T(i,j), for each pair of descriptors by averaging the values, i.e.,
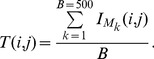
(10)


In order to decide whether the correlation between two descriptors is strong, we choose a cut-off threshold 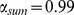. If for the summary-statistic between two descriptors holds the inequality 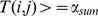, then we define two descriptors to be strongly correlated with each other. The descriptors in the set 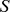 have been chosen as follows. Suppose a descriptor 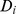 has a maximum number of summary-statistics greater or equal 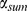 (i.e., 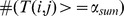, where 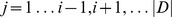), then the descriptor 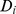 is ranked first, and 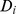 is included in the subset 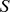. Then we remove the descriptor 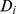 and the other descriptors with which 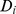 has summary-statistic 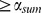. Then, we apply the same procedure to the remaining descriptors until we find any descriptor having maximum number of summary-statistics with remaining descriptors 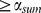. Note that some of the descriptors do not have any summary-statistic greater than 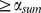 with any of the other descriptors. These descriptors are described as lowly correlated descriptors and such descriptors are also included in the subset 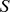.

This procedure reduces 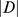 descriptors to 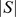 descriptors. That means starting with a set of 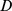 descriptors, we hypothesize that the set 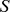 identify structural properties of a graph class distinctly. The remaining 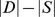 descriptors are showing stronger similarity (correlation) with at least one of the descriptor of set 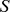.

### Interpretation of the Results

**Figure 1:**
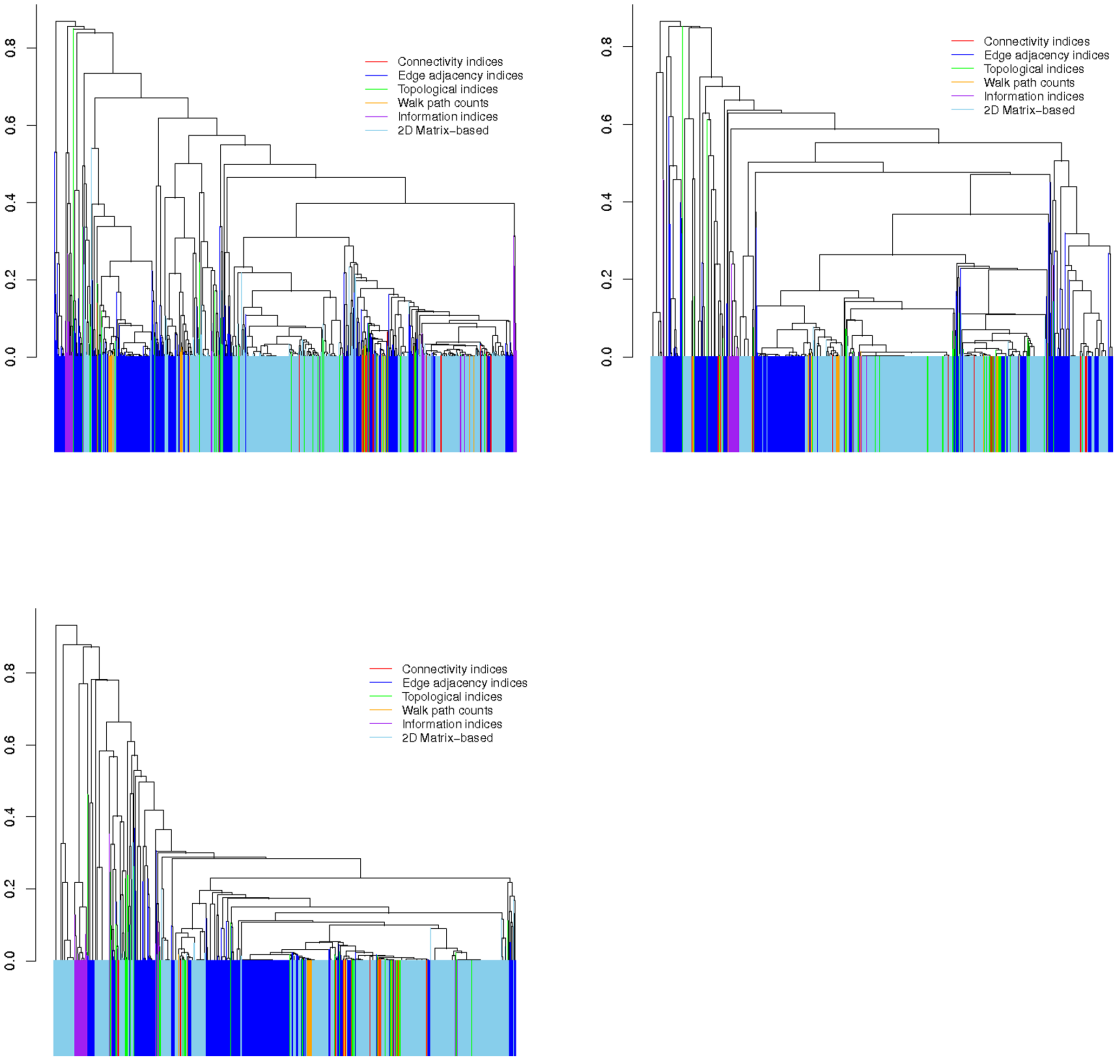
Hierarchical clustering using the average algorithm, 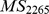 (left), 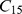 (middle), 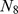 (right). The total number of descriptors equals 919. They belong to 6 different categories which are as follows: connectivity indices (24), edge adjacency indices (301), topological indices (57), walk path counts (28), information indices (40) and 2D Matrix-based (469).

**Figure 2:**
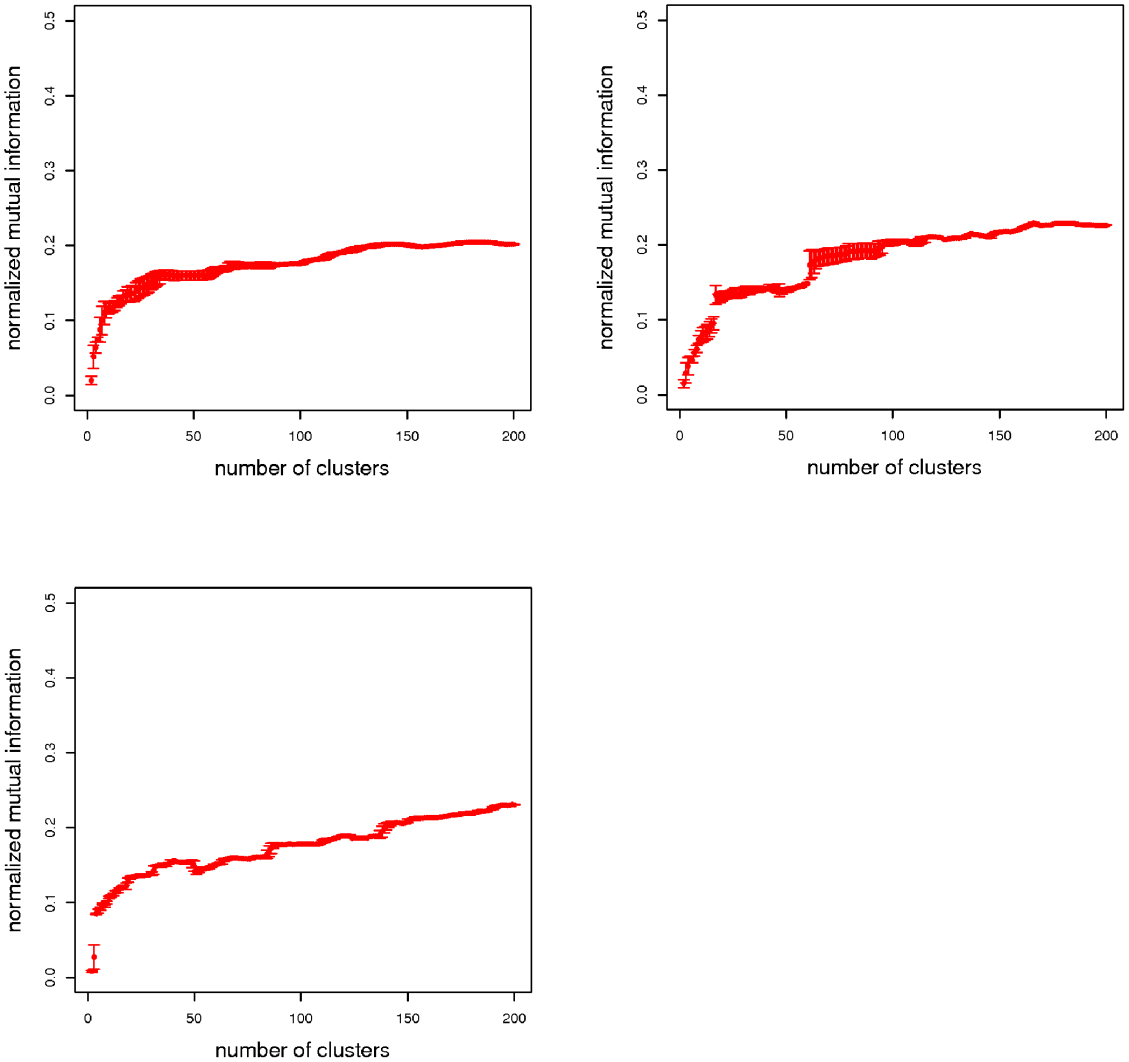
The normalized mutual information, 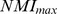, between reference clusters, 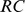, and the number of clusters, 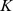, obtained by hierarchical clustering for three data-sets 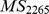 (left), 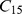 (right) and 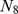 (bottom). 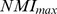 for each 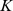 has been generated by sampling the data sets 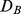, where 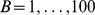 (data set 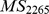). The total number of descriptors equals 919. They belong to 6 different categories which are as follows: connectivity indices (24), edge adjacency indices (301), topological indices (57), walk path counts (28), information indices (40) and 2D Matrix-based (469).

The clustering of descriptors for three datasets is shown by Figure 1. In this figure, the six categories of descriptors are shown in different colors. The figure indicates that the descriptors of each categories have not been clustered correctly regarding their respective groups. For the external validity of the resulting clustering solution, we estimated 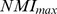 (normalized mutual information) [26] between reference cluster, 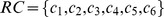 (the descriptors of six categories, 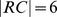, and 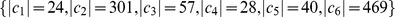 are considered as the groups of the reference cluster) and the number of clusters of the clustering solution by cutting at different heights. The estimated normalized mutual information is calculated by sampling the data 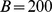 times. Results for the three data-sets (average *NMI*) are shown in Figure 2. The average normalized mutual information plot between the reference cluster and the clusters created by performing average hierarchical clustering shows that they are quite dissimilar, that is the predicted clusters and the reference cluster are not similar at all. Also, the descriptors of different categories are strongly correlated with each other.

**Figure 3:**
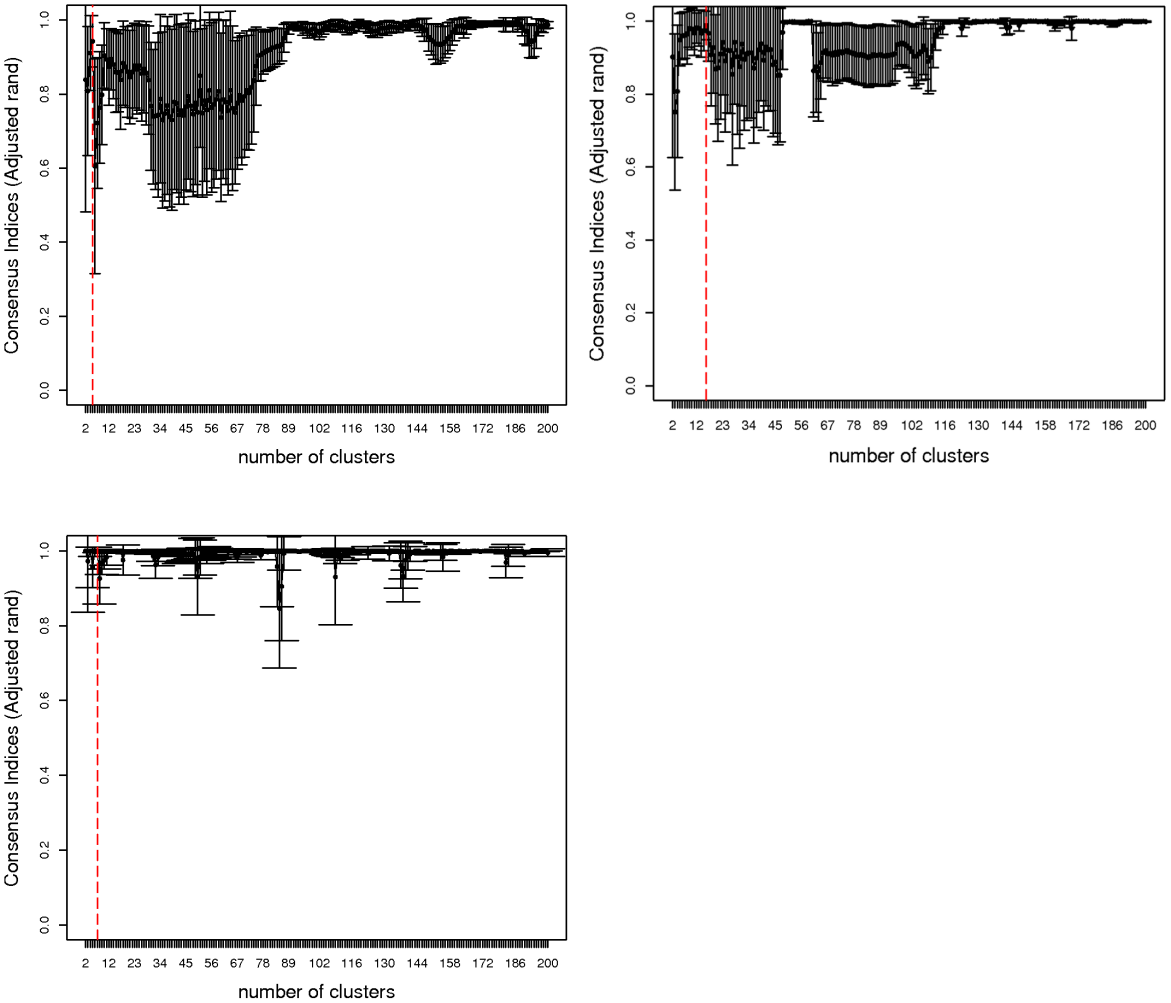
Consensus indices using the *adjusted rand index* for estimating the number of clusters in the data. These plots have been generated by sampling the data sets 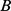, where 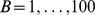 for the three data sets, 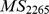 (left), 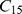 (right), 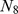 (bottom). The dotted red line shows the optimal number of clusters.

Next, we predict the optimal number of clusters, 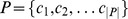 by using consensus indices measure for different number of clusters generated by a clustering solution. The plots for the consensus indices for the three data sets are shown in Figure 3. The consensus indices are calculated for 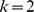, 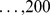 clusters. 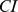 for different number of clusters for the three data-sets does not show an absolute maximum. Therefore we selected the first local maxima which gives the optimal number of clusters. The optimal number of clusters are shown with a dotted red line in the Figure 3. The consensus indices (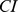) for the optimal number of clusters (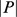) and the total number of descriptors (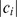, where 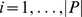) in each cluster for the three data-sets, 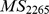, 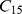 and 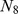 are shown in Table 2. The optimal number of clusters are very little for all three data-sets and for all data-sets. The first cluster is the largest one which contains more than 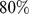 of 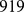 descriptors. The cardinalities of the remaining clusters are smaller as they contain much less descriptors. The largest cluster for all three datasets contains descriptors from all six categories which means that most of the descriptors from different categories have a strong correlation among the descriptors and, therefore, they measure structural information similarly.

**Table 2:** The optimal number of clusters for the three data-sets obtained by using consensus indices (CI).

Data-set	CI	# of clusters ( 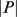 )	# Descriptors in each cluster
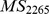	0.942	5	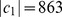 , 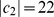 , 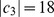 , 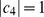 , 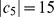
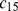	0.9878	16	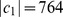 , 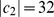 , 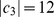 , 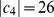 , 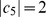 , 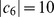 , 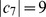 , 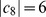 , 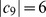 , 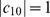 , 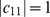 , 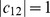 , 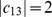 , 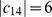 , 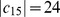 , 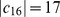
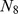	1.00	7	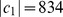 , 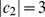 , 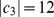 , 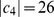 , 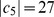 , 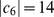 , 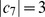

The optimal numbers of clusters (for three data-sets) for a clustering solution 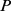 is represented by the set 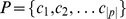, where 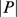 is the optimal number of clusters in the data.

As a next step, we examine the so-called overlap between the optimal number of clusters shown in Table 2 and the six categories of descriptors. That means we have to determine how many different descriptors are distributed over different groups (belonging to the optimal number of clusters). This distribution over different clusters could give some information namely which category might capture structural information of the graphs more uniquely than others. The results are shown in Table 3 and we are going to interpret these results as follows. The intersection of the descriptors between the optimal clusters and the categories of descriptors show that the edge adjacency indices are grouped into different cluster for all three data-sets in comparison to the remaining categories. The 2D Matrix-based descriptors are grouped into different clusters by using 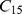 and 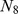. The information indices are grouped into two different clusters by using all three data-sets. The measures from the category walk path counts and topological indices are grouped into different clusters by using 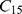 only. This shows that these descriptors behave differently on trees. The overlap indicates that the group of edge adjacency indices contains more descriptors which capture structural information of the graphs differently compared to other categories.

**Table 3:** The descriptors in predicted clusters (rows) overlapping with different categories of descriptors.

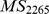						
Number of cluster	connectivity indices	edge adjacency indices	topological indices	walk path counts	information indices	2D Matrix-based
1	24	261	56	28	25	469
2	0	22	0	0	0	0
3	0	18	0	0	0	0
4	0	0	1	0	0	0
5	0	0	0	0	15	0
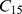						
1	17	214	51	22	34	426
2	4	21	3	2	0	2
3	3	6	1	2	0	0
4	0	26	0	0	0	0
5	0	2	0	0	0	0
6	0	10	0	0	0	0
7	0	9	0	0	0	0
8	0	6	0	0	0	0
9	0	6	0	0	0	0
10	0	1	0	0	0	0
11	0	0	1	0	0	0
12	0	0	1	0	0	0
13	0	0	0	2	0	0
14	0	0	0	0	6	0
15	0	0	0	0	0	24
16	0	0	0	0	0	17
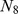						
1	24	287	56	28	14	425
2	0	2	1	0	0	0
3	0	12	0	0	0	0
4	0	0	0	0	26	0
5	0	0	0	0	0	27
6	0	0	0	0	0	14
7	0	0	0	0	0	3

**Figure 4:**
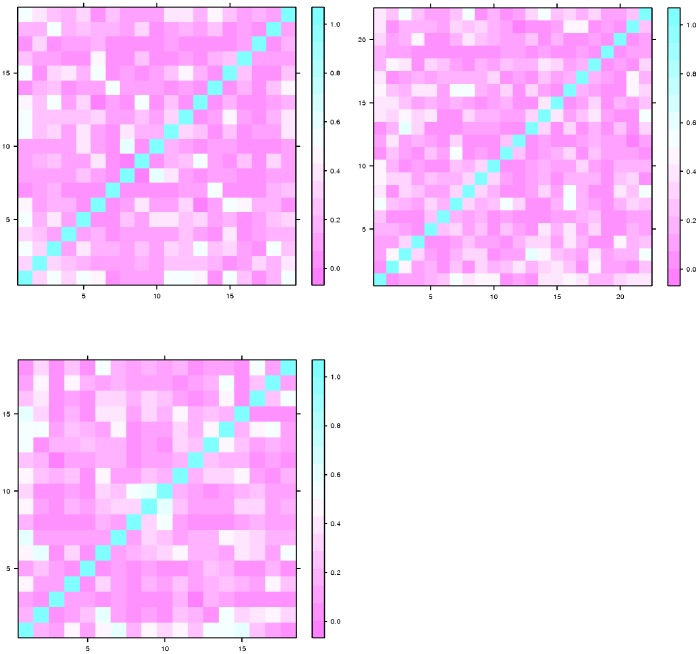
Levelplot of the correlation between the subset 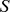 for the three data sets, 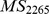 (left), 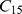 (right), 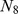 (bottom).

Next, we find a subset of descriptors 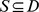, 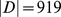. The main idea is to find a smaller set of descriptors which are little correlated with each and, hence, those graph measures captures structural information uniquely. If they would be strongly correlated, they would capture similar structural information of the graphs. Importantly, the remaining descriptors have much stronger correlation with them. The procedure to obtain a subset of descriptors 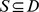 is described in the section 'Methods and Results'. We obtained 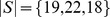 for 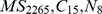 datasets shown in Table 4. The levelplot of correlation for the subset of descriptors of three data-sets are shown in Figure 4. For all three data-sets, we can clearly see that the descriptors of these subsets are not strongly correlated. These subset of descriptors for all three data-set might detect structural features of the molecular graphs uniquely.

**Table 4:** Given the subset 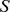; then, the remaining 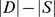 descriptors have at least one pair for which the summary statistic 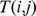 is greater than 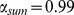 with 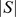 descriptors.

Data-set	Names of the descriptors
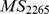	SM3_L, H_Dt, AVS_B.v., SM02_EA.dm., Eig11_AEA.bo., SpMAD_AEA.ed., CIC2, Eig13_AEA.bo., AVS_B.s., SM06_AEA.dm., Eig14_AEA.dm., MAXDP, J_Dz.v., BIC4, SpDiam_AEA.dm., SpMAD_X, PJI2, SpPosA_B.m., IDDE
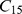	SM2_B.s., PW4, Chi1_EA.ri., SM02_EA.dm., VE1_A, IC2, CENT, SM13_AEA.bo., Eig03_EA.bo., SM03_AEA.dm., VE3_Dz.p., piPC05, Eig04_AEA.bo., SpDiam_AEA.dm., piPC06, Eig02_AEA.dm., IVDE, MAXDP, PJI2, Eig05_AEA.dm., Chi0_EA.dm., Eig07_AEA.ed.
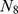	QW_L, TIE, VE3_B.i., BIC1, VE3_Dz.i., Eig10_AEA.dm., SpPosLog_B.m., SM03_AEA.dm., Eig11_AEA.ri., SM04_AEA.dm., CSI, VE1_Dt, Eig08_EA.ed., SpMaxA_AEA.bo., Yindex, Ram, IVDE, Chi1_EA.dm

Moreover we now examine for all data-sets which descriptors from 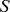 (shown in Table 4) belong to which group out of the six categories of descriptors. The results are summarized in Table 5. For each data-set, we start with a different number of descriptors for the different categories. The subset 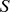 does not contain any descriptor from the *connectivity indices* for all three data-sets, however, only two descriptors from *walk path counts* are contained in 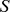 by using 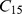. Two, four and three descriptors from the category *topological indices* are contained in 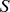 for all three data-sets. Three, two and three descriptors from the category *information indices* are in 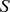 for three data-sets. Seven, three and three descriptors from the category *2D Matrix-based* are in 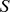 for three data-sets. Seven, eleven and seven descriptors from the category *edge adjacency indices* are in 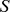 for 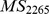, 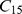, 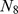. These are the maximal numbers of descriptors compared to other categories of descriptors. The large occurrence of the descriptors from the category *edge adjacency indices* shows again that these descriptors quantify structural information more uniquely than others.

**Table 5:** The number of descriptors of 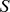 which belong to six different categories by using three data sets.

Descriptor category	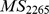	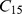	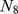
Connectivity indices	0	0	0
Edge adjacency indices	7	11	7
Topological indices	2	4	3
Walk path counts	0	2	0
Information indices	3	2	3
2D Matrix-based	7	3	5

Also, we examine the overlap between the descriptors from 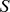 and the descriptors in the found clusters; the intersections between them are shown in Table 6. Interestingly, at least one descriptor (for all data-sets) overlap with the descriptors of each cluster, except for the ninth cluster by using 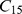. The overlap with the found clusters show that the measures contained in 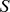 (for three data-sets) have the potential to quantify unique structural features of molecular graphs.

**Table 6:** The overlap between 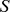 and the predicted clusters (rows).

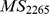	
Number of cluster	Descriptors of 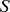
1	SpMAD_AEA.ed., SpDiam_AEA.dm., Eig13_AEA.bo., Eig14_AEA.dm., MAXDP, IDDE, SM3_L, SpMAD_X, H_Dt, J_Dz.v., SpPosA_B.m., AVS_B.v., AVS_B.s.
2	SM02_EA.dm.
3	SM06_AEA.dm., Eig11_AEA.bo.
4	PJI2
5	CIC2, BIC4
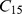	
1	SpDiam_AEA.dm., Eig03_EA.bo., Eig07_AEA.ed., Eig02_AEA.dm., PW4, IC2, SM2_B.s.
2	Chi1_EA.ri.
3	CENT, piPC05
4	SM02_EA.dm.
5	Chi0_EA.dm.
6	Eig04_AEA.bo.
7	SM13_AEA.bo.
8	SM03_AEA.dm.
9	_
10	Eig05_AEA.dm.
11	PJI2
12	MAXDP
13	piPC06
14	IVDE
15	VE1_A
16	VE3_Dz.p.
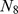	
1	Eig08_EA.ed., Eig10_AEA.dm., Eig11_AEA.ri., CSI, TIE, Yindex, QW_L, SpMaxA_AEA.bo., IVDE, SpPosLog_B.m.
2	Chi1_EA.dm., Ram
3	SM03_AEA.dm., SM04_AEA.dm.
4	BIC1
5	VE3_B.i.
6	VE3_Dz.i.
7	VE1_Dt

## Summary and Conclusions

In this paper, we have evaluated 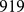 Dragon descriptors to investigate to what extent these measures quantify structural information of molecular graphs uniquely. From our analysis, it is clear that measures which are strongly correlated are not useful as they capture structural information similarly. From this, the question of determining the usefulness or quality of topological indices arises.

We found by calculating the information-theoretic quantity *NMI* that the used six categories of descriptors are strongly correlated with other categories of descriptors. This indicates that despite being categorized into different groups, these descriptors are providing similar information. From this, one can conclude that many of them they have been introduced in an unconsidered manner. Again, the question how useful such indices are seems to be quite important and deserves further attention.

By using all three data sets, the most suitable descriptor subset 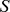 contains those measures which have the largest number of significant correlations with the remaining descriptors but they are not significantly correlated with each other. 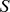 forms a reduced set of descriptors (the original sets contains 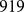 descriptors) and their sizes are feasible approximations of the effective dimension of the descriptor space by using all three datasets. For each individual data set, we found the size of 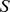 to be 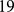 (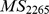 dataset), 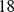 (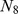 dataset) and 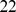 (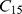 dataset). Because most of the descriptors we have used are redundant, i.e., they are highly correlated, the estimation of the effective dimension is an intriguing problem. In our context, the dimension is the number of different descriptors among all. By performing our analysis, we obtained a lower bound on the dimension of descriptors space regarding the different classes. Note that these descriptors (the ones in 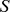) depend on the used data set. By inspecting these subsets, we see that the majority thereof are from the category of the edge-adjacency indices. This implies that the edge-adjacency based descriptors can capture more structural diversity when quantifying structural properties of molecular graphs. As another result of this paper, we see that it would not be appropriate to select descriptors more or less randomly for QSAR problems. Neither the random selection nor using all available descriptors would be appropriate as demonstrated in our paper. To tackle this problem, we suggested a statistical analysis evidenced by using clustering. Again, we note that our method applied to six categories of descriptors reduces the descriptor space for three datasets. In this paper we have presented a statistical approach by using correlation test to select a smaller subset of descriptors which captures information similarly. By employing bootstrapping and a probabilistic measure for the selection process, we have identified the most informative set of descriptors. As seen, a set of descriptors can cover a dataset best, but studying this important issue in depth might be future work.
